# Endothelial dysfunction: The possible link between cardiovascular comorbidities and phenomenon of inflammaging from COPD

**DOI:** 10.1097/MD.0000000000030078

**Published:** 2022-08-19

**Authors:** Emanuela Tudorache, Ovidiu Fira-Mladinescu, Daniel Traila, Monica Marc, Ruxandra Mioara Rajnoveanu, Doina Ecaterina Tofolean, Ariadna Petronela Fildan

**Affiliations:** a Center for Research and Innovation in Personalized Medicine of Respiratory Diseases, XIII^th^ Department – Pulmonology Discipline, “Victor Babes” University of Medicine and Pharmacy Timisoara, Timișoara, Romania; b I^st^ and II^nd^ Clinic of Pulmonary Diseases, Clinical Hospital of Infectious Diseases and Pneumophthisiology “Dr. Victor Babes” Timisoara, Timisoara, Romania; c Palliative Care Department, “Iuliu Hatieganu” University of Medicine and Pharmacy, Cluj-Napoca, Romania; d III^rd^ Department of Internal Medicine, Faculty of Medicine, Ovidius University of Constanta, Constanta, Romania.

**Keywords:** aging, endothelin 1, tumor necrosis factor alpha, vascular inflammation

## Abstract

Aging is a risk factor for many chronic noncommunicable diseases, including chronic obstructive pulmonary disease (COPD), which is often associated with cardiovascular disease (CVD). Moreover, aging is associated with a mild form of systemic inflammation. The aim of our study was to analyze the relationship between age, systemic and vascular inflammation, and the presence of CVD comorbidities in a stable COPD population. Forty COPD patients were divided into 2 age groups (<65 and ≥65 years of age), from which we collected the following inflammatory biomarkers: C-reactive protein, tumor necrosis factor alpha (TNF-α), interleukin-6 (IL-6), and endothelin-1 (ET-1). Elderly COPD patients had more frequent exacerbation events per year (2 vs 1, *P* = .06), a higher prevalence of CVD (3 vs 2, *P* = .04), more limited exercise tolerance (6-minute walking test distance, 343 [283–403] vs 434 [384–484]; *P* = .02), and mild systemic inflammation (TNF-α, 9.02 [7.08–10.96] vs 6.48 [5.21–7.76]; *P* = .03; ET-1, 2.24 [1.76–2.71] vs 1.67 [1.36–1.98] pg/mL; *P* = .04). A weak correlation between age and ET-1 (*r* = 0.32, *P* = .04) was observed. Mild systemic inflammation, characterized by a slightly increased level of TNF-α, and endothelial dysfunction, marked by elevated ET-1, could be liaisons between aging, COPD, and CVD comorbidities.

## 1. Introduction

Worldwide, we are witnessing a rise in life expectancy, which is associated with an increased incidence of chronic obstructive pulmonary disease (COPD) and other noncommunicable age-related diseases.^[1,2]^ Physiological aging is characterized by a progressive decline in all vital organ functions, including lung function.^[1]^ The aging process is correlated with mild systemic inflammation. Inflammatory biomarkers are independent predictors of mortality in older adults. Proinflammatory cytokines such as tumor necrosis factor alpha (TNF-α), interleukin 6 (IL-6), and interleukin 8 (IL-8) are thought to induce senescence in adjacent cells.^[3]^

COPD is characterized by persistent, progressive airflow limitation caused by a chronic inflammatory response of the lungs to noxious particles and gases. Systemic manifestations, comorbidities, and acute exacerbations influence the morbidity and mortality of this disease.^[4]^ It is well known that systemic inflammation is common during COPD exacerbation periods, but recently, it has been reported that some stable COPD patients also associate a chronic, mild form of systemic inflammation. This particular type of inflammation is characterized by a slight elevation of inflammatory biomarkers, such as fibrinogen, C-reactive protein (CRP), TNF-α, and IL-6.^[5–7]^

Cardiovascular diseases (CVDs) are the most common COPD comorbidities and have a significant impact on disability and mortality. The relationship between COPD and CVD partly be explained by the presence of common risk factors such as smoking history, sedentary lifestyle, and the aging process.^[3,5,8,9]^ COPD makes the cardiovascular system more vulnerable, particularly the endothelium, favoring the development and destabilization of atherosclerotic plaques, particularly in elderly patients (e.g., >65 years of age). Consequently, mortality caused by CVD accounts for 25% of all causes of death in patients with COPD.^[7,10]^

On the molecular level, endothelins are implicated in vascular diseases of several organ systems, including heart, brain, lungs, and kidneys, and they play a key role in vascular system homeostasis.^[11,12]^ Endothelin-1 (ET-1) is considered the parent compound of the endothelin family, comprising 21 amino acids with a free amino terminus and C-terminal carboxylic acid.^[13]^ ET-1 is the most abundant isoform in the human cardiovascular system, and the primary source is thought to be vascular endothelial cells, although the peptide is also produced by other cell types, including epithelial cells, such as the lungs, kidney, and colon.

Given these factors, this study was aimed to evaluate the impact of aging on stable COPD patients in terms of systemic and vascular inflammatory biomarkers (CRP, TNF-α, IL-6, and ET-1) and cardiovascular comorbidities.

## 2. Materials and Methods

### 2.1. Case selection

This was a cross-sectional study that included COPD in patients who were routinely investigated, monitored at regular intervals, and followed up in our hospital. The study was approved by the ethical committee of the Hospital of Infectious Diseases and Pneumophthisiology “Dr Victor Babes,” Timisoara, Romania. The inclusion criteria were the following: patients with a confirmed diagnosis of COPD in a stable disease phase (according to European Respiratory Society/American Thoracic Society guidelines), a history of former smoking ≥10 pack-years, and the ability to provide informed consent. The exclusion criteria were as follows: acute respiratory failure, asthma-COPD overlap, pulmonary embolism, active infections, acute CVD, malignancy, recent surgery, severe endocrine, hepatic or renal diseases, autoimmune diseases, and inflammatory disorders (i.e., polyarthritis, chronic inflammatory bowel disease, and systemic lupus erythematosus). To exclude confounders that might lead to systemic inflammation, current smokers were also excluded.

All eligible patients with stable COPD (clinically stable airway obstruction that did not require any change in therapeutic treatment plan in the last 3 mo) signed an informed consent form before entering the study. There were no invasive interventions or treatment modifications for the purpose of this study. Forty patients with stable COPD were included and divided by age into 2 groups: group A, 19 patients aged <65 years, and group B, 21 patients aged >65 years. No dropouts occurred. On the first day of hospital admission, the patients’ personal medical history and the number of exacerbation events in the last 12 months were recorded.

### 2.2. Biological and functional parameters of the patients

Blood samples, proinflammatory biomarkers (CRP, TNF-α, and IL-6), and ET-1 were collected from all subjects after a 12-hour fast. Serum was obtained after centrifugation at 1500 rpm for 15 minutes and stored at −80 °C until assayed. These biomarkers were assessed according to the manufacturer’s instructions using high-sensitivity kits, and an enzyme-linked immunosorbent assay technique was performed (Endothelin-1 [Quantikine Enzyme-Linked Immunosorbent Assay Kit, R&D Systems Inc., Minneapolis, MN, Catalog Number DET100]; TNF-alpha [Immulite Siemens Healthcare Diagnostics Products Ltd., Glyn Rhonwy, Llanberis, Ðwynedd, United Kingdom, Catalog Number LKNF1]; IL-6 [Elecsys IL-6, Cobas, Roche Diagnostics, Mannheim, Germany. GmbH, Germany; catalog number 05109442 190]). Normal values for these assays were considered below 2 pg/mL for ET-1, 8.1 pg/mL for TNF-α, and 7 pg/mL for IL-6. A Jaeger spirometer was used to test lung function. Spirometry was used for COPD diagnosis and follow-up. The classification of airflow limitation severity in COPD is based on the value of Forced Expiratory Volume in the first second (FEV1): FEV1 >80% (mild COPD), FEV1 = 50%–80% (moderate), FEV1 = 30%–50% (severe), and FEV1 <30% (very severe).^[4]^ According to the ATS/ERS statement, the forced vital capacity (FVC), FEV1, and ratio FEV1/FVC were measured 3 times, and the best value was reported. Using a shutter, we also assessed maximal expiratory pressure (MEP) and maximal inspiratory pressure (MIP). Subjects’ body composition (body fat, soft lean mass [SLM], and Soft Lean Mass Index) were analyzed using a Biodynamics BIA 310e Bioimpedance Analyzer, Biodynamics Corporation, Shoreline, WA.

Another useful tool used for the global evaluation of patients with COPD is the modified Medical Research Council (mMRC) dyspnea scale score, which evaluates the severity of dyspnea, since a score >2 is related to an increased risk of mortality in this population.^[4]^

Electrocardiogram and echocardiography (Siemens Medical Solution, Erlangen, Germany) were performed by the same cardiologist on the second day of admission and were correlated with patients’ personal medical history. Most common CVD registered were hypertension, atherosclerosis/ischemic heart disease, heart failure, cardiac arrhythmias, pulmonary hypertension, and peripheral arterial disease.

The 6 minutes walking distance (6MWD) is a simple tool for evaluating the exercise capacity of patients with chronic pulmonary diseases. The results are correlated with the optimal reference equation for healthy individuals. Results under 80% were associated with poor COPD prognosis.^[1]^

Patients completed the COPD Assessment Test (CAT) questionnaire and performed the 6 minutes walking test, according to the European Respiratory Society/American Thoracic Society statement, under the supervision of a physiotherapist. The CAT is an 8-item questionnaire that evaluates health status impairment in COPD. A total score below 10 points indicates an acceptable health status, while a score over 10 points is a warning sign for the burden of COPD on the patient’s health status.^[4]^

The number of moderate/severe exacerbations registered in the last 12 months was a good predictor of the risk of future exacerbations. More than 1 exacerbation/year correlates with a worse outcome.^[4]^ Combined COPD assessment is mandatory to understand the impact of the disease on individuals. It includes the degree of airflow obstruction, CAT, mMRC, and the number of moderate/severe exacerbations per year. This comprehensive assessment is considered a good predictor of clinical outcomes and survival rates in the COPD population.^[4]^

### 2.3. Statistical analysis

Data were collected and analyzed using GraphPad Prism 7, GraphPad Software Inc., San Diego, CA and are presented as mean with 95% confidence intervals for continuous variables with a Gaussian distribution, respectively, median, and interquartile range for continuous variables without a Gaussian distribution. Continuous variable distributions were tested for normality using the Shapiro-Wilk test and if *P* >.05, and Gaussian distribution was assumed. To evaluate the significance of the differences between groups, unpaired Student (means, Gaussian populations) and Mann-Whitney (medians, non-Gaussian populations) tests were used. Frequencies were compared using the chi-square test. To evaluate the association between two quantitative variables, a Pearson coefficient of correlation was computed if the variables were in a linear relationship and with Spearman coefficient of correlation if not. A *P* value <0.05 was considered the threshold for statistical significance. The datasets used and/or analyzed during the current study are available from the corresponding author upon reasonable request.

## 3. Results

The patient characteristics for the 2 age groups and the results of the comparative analysis are presented in Table 1. Compared to younger patients, the elderly group had an increased number of CVD comorbidities (CVD, 3 [2–4] vs 2 [1–3]; *P* = .04), more COPD exacerbations/year (2 [1–2] vs 1 [0–2]; *P* = .06), more limited effort capacity (6MWD, 343 [283–403] vs 434 [384–484]; *P* = .02), and a slightly higher dyspnea score (mMRC, 3 [2–4] vs 3 [1–3]; *P* = .05). All patients were former smokers with a personal history of heavy smoking in both groups above 30 pack-years (*P* = ns).

**Table 1:** Clinical characteristics and physiology of patients.

Parameters	<65 yr group A	≥65 yr group B	*P* value
Male/female (n)	13/6	17/4	.361
Smoking (pack years)	30 (20–70)	40 (22.5–67.5)	.446
CV comorbidities (n)	2 (1–3)	3 (2–4)	**.042** *
Exacerbations per year (n)	1 (0–2)	2 (1–2)	.061
Exacerbators vs nonexacerbators (n)	9/10	14/7	.218
CAT score	21 (10–25)	20 (14.5–22.5)	.804
mMRC score	3 (1–3)	3 (2–4)	.052
SBP (mm Hg)	125 (117–132)	134 (126–142)	.078
DBP (mm Hg)	76 (71–80)	78 (72–83)	.562
FVC (% pred.)	65.71 (56.58–74.84)	64.43 (56.35–72.51)	.826
FEV1 (% pred.)	42.64 (31.75–53.53)	45.67 (37.68–53.66)	.637
FEV1/FVC	0.53 (0.45–0.60)	0.57 (0.51–0.62)	.345
BMI (kg/m^2^)	26.23 (22.81–29.64)	25.95 (23.78–28.13)	.886
BF (%)	22.88 (17.58–28.19)	25.16 (20–30.31)	.524
SLM (kg)	52.61 (45.01–60.21)	46.41 (39.3–53.52)	.220
SLMI (kg/m^2^)	20.06 (17.45–22.67)	15.84 (12.31–19.37)	**.048** *
MIP (cm H_2_O)	68.3 (56.9–79.8)	56.9 (47.2–66.6)	.116
MEP (cm H_2_O)	67.9 (55.1–80.7)	58.9 (48.1–69.6)	.262
6MWD (m)	434 (384–484)	343 (283–403)	**.022** *
6MWD (% pred.)	85.17 (76.23–94.11)	80.25 (67.22–93.27)	.527
CRP (mg/L)	4.76 (3.66–5.87)	4.78 (3.98–5.56)	.987
IL-6 (pg/mL)	6.39 (5.07–7.71)	7.74 (6.82–8.66)	.082
TNF-α (pg/mL)	6.48 (5.21–7.76)	9.02 (7.08–10.96)	**.032** *
ET-1 (pg/mL)	1.67 (1.36–1.98)	2.24 (1.76–2.71)	**.047** *

Data are presented as mean with 95% confidence interval for parametric values and median with interval from 25% to 75% percentiles for nonparametric data.

6MWD = 6 minutes walking test distance, BF = body fat, BMI = body mass index, CAT = COPD Assessment Test, CRP = C-reactive protein, CV = cardiovascular, DBP = diastolic blood pressure, ET-1 = endothelin 1, FEV1 = Forced Expiratory Volume in the 1 second, FVC = forced vital capacity, IL-6 = interleukin 6, MEP = maximal expiratory pressure, MIP = maximal inspiratory pressure, mMRC = modified Medical Research Council, SBP = systolic blood pressure, SLM = soft lean mass, SLMI = soft lean mass index, TNF-α = tumor necrosis factor alpha.

* A *P* value <.05 was considered the threshold for statistical significance.

There were no differences between the 2 groups regarding the severity of airflow obstruction (FEV1, 42.64 [31.75–53.53] vs 45.67 [37.68–53.66]% pred; *P* = ns), MIP and maximal expiratory pressure (MIP, 68.3 [56.9–79.8] vs 56.9 [47.2–66.6] cm H_2_O; *P* = ns; MEP, 67.9 [55.1–80.7] vs 58.9 [48.1–69.6] cm H_2_O; *P* = ns), or body mass index (BMI, 26.23 [22.81–29.64] vs 25.95 [23.78–28.13] kg/m^2^; *P* = ns).

Elderly patients presented a mild grade of systemic inflammation, with statistically significant elevation of some inflammatory biomarkers (TNF-α, 6.48 [5.21–7.76] vs 9.02 [7.08–10.96] pg/mL; *P* = .03; ET-1, 1.67 [1.36–1.98] vs 2.24 [1.76–2.71] pg/mL; *P* = .04), whereas others were only marginally elevated (IL-6, 6.39 [5.07–7.71] vs 7.74 [6.82–8.66] pg/mL; *P* = .08) or without significant elevation (CRP, 4.76 [3.66–5.87] vs 4.78 [3.98–5.56]; *P* = ns).

**Figure 1.**
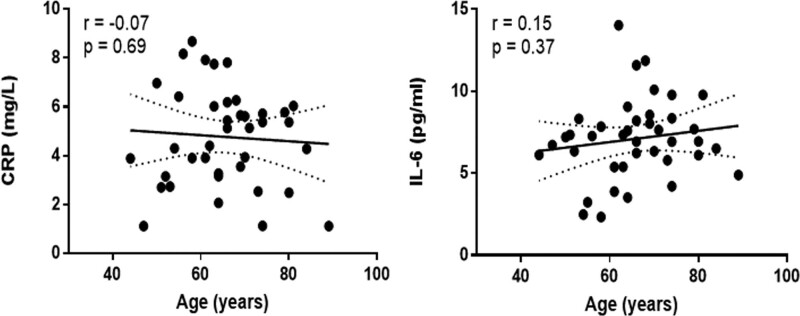
Age correlation with CRP and IL-6 in COPD patients. COPD = chronic obstructive pulmonary disease, CRP = C-reactive protein, IL-6 = interleukin-6.

**Figure 2.**
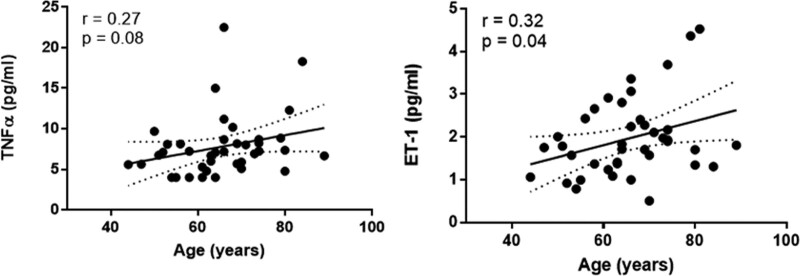
Age correlation with TNFα and ET-1 in COPD patients. COPD = chronic obstructive pulmonary disease, ET-1 = endothelin-1, TNFα = tumor necrosis factor alpha.

Among the analyzed biomarkers, we observed a weak correlation with age (Figs. 1 and 2) only for ET-1, which was also statistically significant (*r* = 0.32, *P* = .04), and TNF-α, but the difference was not statistically significant (*r* = 0.27, *P* = 0.08).

**Figure 3.**
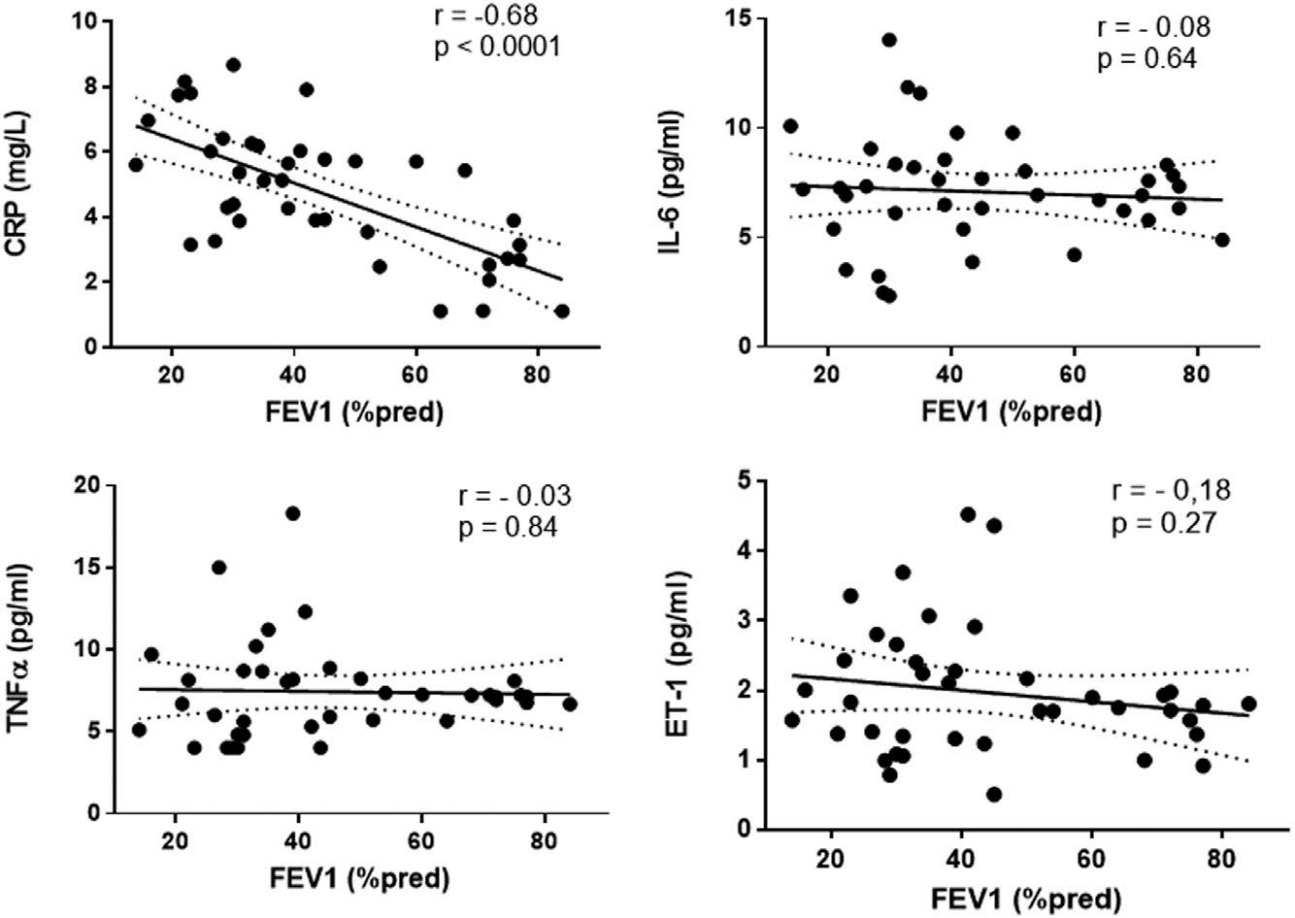
FEV1 (%) correlations with analyzed biomarkers in COPD patients: CRP; IL-6; TNF-α; ET-1. COPD = chronic obstructive pulmonary disease, CRP = C-reactive protein, ET-1 = endothelin-1, FEV1 = Forced Expiratory Volume in the first second, IL-6 = interleukin-6, TNF-α = tumor necrosis factor alpha.

On the other hand, the severity of COPD (according to FEV1) significantly correlated with CRP levels (*r* = −0.68, *P* < .0001), but not with TNF-α (*r* = −0.03, *P* = .84), IL-6 (*r* = −0.08, *P* = .64), or ET1 (*r* = −0.18, *P* = .27) (Fig. 3).

## 4. Discussion

Our results have shown that in older patients with COPD, biomarkers of systemic inflammation and endothelial dysfunction are more highly expressed, and there is a direct correlation between the patient age and vascular inflammation. This observation is supported by researchers who named this phenomenon as “inflammaging,” considering that this inflammatory process contributes to the development and progression of a large variety of chronic pathologies like COPD, CVD, diabetes, metabolic syndrome, muscle weakness, depression and cognitive impairment, etc., in elderly populations.^[6,14–16]^ According to Agusti et al,^[6]^ smoking itself, without obstructive airflow limitation, is correlated with a mild form of inflammation, especially with an increased level of TNF-α. Accordingly, to eliminate possible confounders, active smokers were excluded from our study. We found that COPD severity (FEV1) significantly correlated with CRP levels, but not with TNF-α. Su et al^[17]^ performed a systematic review and meta-analysis, which included 24 observational studies on 10,677 COPD patients. Their results showed that COPD was associated with elevated CRP, IL-6, IL-8, leukocyte, and fibrinogen levels, without statistically significant differences in TNF-α levels. In this context, our results suggest that TNF-α is rather a mediator of inflammaging than of COPD-specific inflammation.

Similar to asthma, older COPD patients, despite having similar lung function limitations, tend to be more symptomatic and have a higher prevalence of exacerbations, cardiovascular morbidity, and muscular impairment.^[5–7]^ From this perspective, the exclusion criteria in our study included bronchial asthma with adult-onset, which can mimic COPD, especially among smokers.^[18]^

There are several common risk factors that can lead to CVD in the COPD population, including smoking history, hypoxia, sedentariness, aging, and systemic inflammation.^[6,19]^ It is well known that decreased FEV1 is correlated with an increased risk of atherosclerosis; therefore, we can assume that endothelial dysfunction could mediate the relationship between COPD and CVD.^[20]^ Endothelial injury is linked to emphysema development, while endothelial dysfunction is linked to systemic inflammation and oxidative stress. Both diseases commonly develop in COPD patients. Recently, it has been suggested that endothelial cells are more involved than alveolar epithelial cells in alveolar gas exchange and have a greater impact on COPD pathogenesis.^[21]^ ET-1 is a marker of endothelial dysfunction that acts as a vasoactive mediator, leading to vasoconstriction and vascular wall proliferation.^[20,22]^ In smokers, accelerated lung aging, influenced by cigarette smoking, plays an important role in the development of COPD. Previous studies have found an association between increased baseline CRP and airway obstruction severity, a higher number of exacerbations, and a higher risk of early mortality.^[23,24]^ Our study showed that COPD severity was associated with increased CRP levels but did not significantly correlate with ET-1. However, among our elderly patients with COPD, ET-1 blood levels were significantly increased. Therefore, could we consider an endothelial disorder, as researchers have recently revealed?^[21]^ CRP, which is released as a result of vascular damage, stimulates IL-6 and ET-1 production. Thus, an increased CRP level can be considered an independent risk factor for CVD and is associated with worse cardiovascular outcomes in COPD patients. In the CV system, IL-6 stimulates the atherosclerotic process and promotes frequent exacerbations and rapid decline of the lung function within the respiratory system.^[18,25,26]^ According to other authors, IL-6 modulates the relationship between aging and chronic non-communicable diseases such as CVD, COPD, and diabetes.^[25]^

Systemic inflammation in COPD also correlates with respiratory and peripheral muscle weakness and reduced physical activity.^[27–30]^ We found that older patients, most of whom presenting elevated proinflammatory biomarkers, tended to experience respiratory muscle weakness, although the difference between the analyzed parameters (MIP and MEP) was not statistically significant. Other authors analyzed the changes in physical activity in elderly individuals with/without COPD, revealing a significant decrease in physical activity in both categories; however, COPD patients registered worse results, corresponding to their disease severity.^[30]^ Although both groups experienced limited exercise tolerance in the 6MWD test, elderly patients were more affected. Furthermore, because there was no difference between the two groups regarding the 6 minutes walking test (%), this suggests that the phenomenon of aging is responsible for the decline in functional status. This finding is consistent with those of other studies that indicate age as an independent risk factor for decreased physical activity in patients with COPD.^[31,32]^ The study of Corlateanu et al^[31]^ revealed a significant correlation between TNF-α and the 6MWD test. Moreover, airflow obstruction predicted a higher level of IL-6, which, in turn, determined a worse 6MWD score.^[31]^

Obesity and metabolic syndrome are the major determinants of systemic inflammation in the general population. Some studies have shown that an increased BMI is associated with more severe airflow obstruction, systemic inflammation, CVD, diabetes, and sleep apnea.^[6,14,32–34]^ On the other hand, a lower BMI can be an independent negative prognostic factor in COPD patients. Sarcopenia is characterized by a low SLM and is a strong predictor of peripheral muscle weakness associated with reduced exercise capacity and high mortality.^[35,36]^ Moreover, elevated IL-6, IL-8, and TNF-α levels have been associated with advanced muscle loss, malnutrition, and cachexia.^[36]^ Although there was no apparent difference in body composition between our groups, SLM was lower in elderly patients (SLM: 20.06 vs 15.84 kg/m_2_, *P* = .04), suggesting a degree of sarcopenia in group B.

We do acknowledge several limitations of this study. First, it was a retrospective observational study conducted at a single center; however, its conclusions could be a reliable starting point for further prospective and multicenter research studies. Second, the small sample size of our study limited the discrimination of age as a risk factor for systemic inflammation, endothelial dysfunction, and limited exercise tolerance. Moreover, the conclusions of the study were based only on the data at which the statistical analysis proved to be significant. However, the panel of analyzed biomarkers is not commonly used in current clinical practice, and the costs involved in the acquisition of reagents are the main limiting factors of the study group size. Finally, since inflammatory biomarkers are common in CVD, further studies focusing on the comparison between younger and older COPD patients without CVD should be performed.

## 5. Conclusions

In a stable COPD population, aging is associated with a mild grade of systemic inflammation, endothelial dysfunction (elevated ET-1), increased prevalence of cardiovascular comorbidities, and limited exercise tolerance. Endothelial dysfunction may be a link between COPD, aging, and cardiovascular comorbidities. Elevated ET-1 level is a biomarker of endothelial dysfunction and should be monitored in COPD, especially in older patients with cardiovascular comorbidities. This observation of endothelial dysfunction in the elderly population with COPD could have important therapeutic implications.

## Acknowledgments

The funds necessary for acquisition of reagents came from a doctoral grant supported by “Victor Babes” University of Medicine and Pharmacy Timisoara.

## Author contribution

Conceptualization, E.T., O.F.M., and A.P.F.; methodology, O.F.M.; software, D.T.; validation, E.T., A.P.F., and O.F.M.; formal analysis, M.M., R.M.R., and D.E.T.; investigation, E.T.; data curation, D.E.T.; writing-original draft preparation, E.T.; writing-review and editing, O.F.M., D.T., and A.P.F.; visualization, M.M, R.M.R., and D.E.T.; supervision, A.P.F. All authors have read and agreed to the published version of the article.
